# Nrp-1 Mediated Plasmatic Ago2 Binding miR-21a-3p Internalization: A Novel Mechanism for miR-21a-3p Accumulation in Renal Tubular Epithelial Cells during Sepsis

**DOI:** 10.1155/2020/2370253

**Published:** 2020-08-18

**Authors:** Zhiqiang Zou, Qin Lin, Huobao Yang, Zhongwei Liu, Shixiang Zheng

**Affiliations:** ^1^Department of Critical Care Medicine, Union Hospital of Fujian Medical University, Fuzhou, Fujian, China 350001; ^2^Department of Anesthesiology, Fujian University of Traditional Chinese Medicine affiliated People's Hospital, Fuzhou, Fujian, China 350001; ^3^Department of Critical Care Medicine, Shengli Clinical Medical College of Fujian Medical University, Fujian Provincial Hospital, Fuzhou, Fujian, China 350001; ^4^Department of Cardiology, Shaanxi Provincial People's Hospital, Xi'an, Shaanxi, China 710068

## Abstract

The mechanism underlying sepsis-associated acute kidney injury (SAKI), which is an independent risk factor for sepsis-associated death, is unclear. A previous study indicates that during sepsis miR-21a-3p accumulates in renal tubular epithelial cells (TECs) as the mediator of inflammation and mediates TEC malfunction by manipulating its metabolism. However, the specific mechanism responsible for the accumulation of miR-21a-3p in TECs during sepsis is unrevealed. In this study, a cecal ligation and puncture- (CLP-) induced sepsis rat model and rat TEC line were used to elucidate the mechanism. Firstly, miR-21a-3p and Ago2 levels were found out to increase in both plasma and TECs during sepsis, and the increase of intracellular Ago2 and miR-21a-3p could be mitigated when Ago2 was either inactivated or downregulated in septic plasma. Moreover, membrane Nrp-1 expression of TECs was increased significantly during sepsis and Nrp-1 knockdown also mitigated the rise of both the intracellular Ago2 and miR-21a-3p levels in TECs incubated with septic plasma. Furthermore, it was revealed that Ago2 can be internalized by TECs mediated with Nrp-1 and this process had no effect on the intracellular content of miR-21a-3p. Both Ago2 and miR-21a-3p could bind to TECs derived Nrp-1 directly. Finally, it was determined that miR-21a-3p was internalized by TECs via Nrp-1 and Ago2 facilitated this process. Taken together, it can be concluded from our results that Ago2 binding miR-21a-3p from septic plasma can be actively internalized by TECs via Nrp-1 mediated cell internalization, and this mechanism is crucial for the rise of intracellular miR-21a-3p content of TECs during sepsis. These findings will improve our understanding of the mechanisms underlying SAKI and aid in developing novel therapeutic strategies.

## 1. Introduction

Dysregulated host response to infection results in the development of sepsis, which is the leading cause of death in the intensive care unit (ICU) worldwide [1]. Thus, sepsis is an important public health issue with considerable economic consequences [2]. A new definition which was named sepsis 3.0 was developed in 2016 to further refine this complicated process, with an increased focus on early recognizing and treating organ dysfunction in the context of infection to receive better prognosis [3, 4]. Although the definition of sepsis is constantly being updated to achieve a better clinical prognosis according to relevant clinical features, the mortality of sepsis remains unacceptably high. Moreover, sepsis in patients with pathological conditions, such as chronic obstructive pulmonary disease and coronavirus disease 2019-related pneumonia can exacerbate the disease severity and increase mortality [5–7]. MODS, especially acute kidney injury, induced by sepsis, is the independent risk factor of septic death which related mechanisms remain unclear [8, 9]. Thus, elucidating the mechanism underlying sepsis-associated AKI (SAKI) can aid in developing effective preventive and therapeutic strategies.

Sepsis is reported to promote renal tubular epithelial cell (TEC) injury. Furthermore, TECs undergo malfunctioning rather than suffering from cell death during sepsis [10, 11]. The accumulation of various factors, including microRNAs (miRs), in the TECs mediates the adaptive and/or dysregulated responses, which can lead to cellular dysfunction [12–14]. miRs are a specific group of noncoding RNA that can regulate the expression of target proteins posttranscriptionally via interacting with their mRNAs. Many miRs have been well studied in either animals or human researches and are found out to be involved in the mechanisms of sepsis and related organ dysfunction [15]. miR-21 is expressed in many organs such as the heart and kidney of mammals. Studies have indicated miR-21 involves many kinds of processes such as fibrosis and podocyte apoptosis which can cause kidney injury [16–18]. In our recent study on SAKI, miR-21a-3p was revealed to accumulate in TECs during sepsis and was verified to mediate metabolism and cell fate alteration of TECs via manipulating the AKT/CDK2-FOXO1 pathway. Moreover, this mechanism played a novel role in the regulation of energy metabolism of TECs during SAKI [19]. However, the mechanisms underlying sepsis-induced miR-21a-3p accumulation in the TECs have not been elucidated.

Recent studies have shown a growing consensus that both immune and nonimmune cells can constantly release miRs into an extracellular environment [20, 21]. miRs are found to be extremely stable in the extracellular fluid such as blood plasma and urine when binding to RNA-binding proteins such as Argonaute-2 (Ago2) [22, 23]. In recent years, efforts have been made to reveal the miRs that are differentially expressed in the circulation of sepsis patients and found out that the blood contents of specific miRs were upregulated when sepsis occurred. As a result, other than being recognized to act as biomarkers of some diseases [24, 25], miRs are considered to be “hormones” that regulate cell functions and metabolism in cell-cell communications. Since miR-21a-3p was identified as an important inflammatory factor manipulating TEC metabolism, it is needed to verify that if the plasma miR-21a-3p increased and was uptaken by TECs during sepsis. Furthermore, the relevant mechanisms also need to be demonstrated.

In our present study, relevant experiments on the CLP sepsis rat model and rat renal tubular epithelial cell line were carried out in order to find out the role receptor-mediated cell internalization played in the accumulation of miR-21a-3p in TECs during sepsis and relevant mechanisms.

## 2. Materials and Methods

### 2.1. Animals

Specific pathogen-free Sprague-Dawley rats with an average bodyweight of 200–300 g were procured from the Hubei Institute of Experimental Animal. The relevant study protocols were approved by the Institutional Animal Use and Care Committee of Fujian Medical University Union Hospital. The rats were housed in a laminar flow room under controlled temperature and humidity conditions with an artificial 12 h light/dark cycle. All rats had free access to tap water and standard rat chow. The rats were divided into the control group and cecal ligation and puncture (CLP) group randomly. The specimens of the control group were harvested 12 hours after sham operation. For the CLP group, the specimens were harvested at 12 hours, 18 hours, and 24 hours after the surgery, respectively, for further inspection and study.

### 2.2. Surgical Protocols

CLP was performed as described before. In short, the rat was anesthetized with isoflurane and dissected to expose the cecum. The cecum was punctured in a through-and-through way at the midpoint between the end tip of the cecum and the ligation, which was located at the midpoint of the cecum. A small amount of feces were excluded before closing the abdominal cavity, and prewarmed normal saline (37°C, 5 ml/100 g) was injected subcutaneously for resuscitation. For the sham operation of the control group, the cecum was located and was returned into the abdominal before the abdominal cavity was closed.

### 2.3. TEC Isolation

TECs from the kidney of the rats were isolated as described before. Briefly, the cortex of the kidney was isolated carefully and ground on 80 and 100 mesh screens. The residue on the 100 mesh screen was digested with 0.2% trypsin (Hyclone). After being washed with PBS for 3 times, the TECs were sorted with magnetic cell sorting technique with FITC anti-cytokeratin 18 antibody (Abcam) and anti-FITC microbeads (Miltenyi Biotec).

### 2.4. Cell Treatment

The NRK52E cell line was cultured in Dulbecco's modified Eagle's medium (DMEM) (Hyclone) supplemented with 10% fetal bovine serum and 1% penicillin-streptomycin solution at 37°C and 5% CO_2_. For specific protein expression knockdown groups and their negative controls, relevant siRNAs (Nrp-1 siRNA, siRNA NC (GenePharma)) were transfected to the cells cultured to 70-80% confluence using Lipofectamine™ 2000 according to the manufacturer's instructions (Invitrogen) before further treatments. For different plasma incubations, firstly, the cells of different groups were cultured with the serum-free DMEM for 12 h; then, specific plasma was added accordingly and incubated with the cells for 12 hours. For the miR-21a-3p mimics and/or Ago2 treatments, all the cells with or without transfection were washed 3 times with PBS and cultured for 12 hours in a serum-containing medium; then, the cells were cultured in serum-free DMEM for 12 hours, after that the miR-21a-3p single strain mimics, biotin-labeled miR-21a-3p single strain mimics, FAM-labeled miR-21a-3p single strain mimics (GenePharma), and Ago2 (Sino Biological) were used to incubate the cells in serum-free DMEM accordingly for 12 hours before further inspections. For the Ago2 and miR-21a-3p costimulation, equimolar Ago2 and miR-21a-3p were premixed for 1 hour to form the Ago2/miR-21a-3p mimics complex before use.

### 2.5. Plasma Treatment

Septic plasma was obtained from the sepsis rats euthanized 12 hours after the CLP operation. Ago2 monoclonal antibody (Novus) at the final concentration of 150 pg/ml was added to the septic plasma with or without further immunoprecipitation to form either Ago2 immunoprecipitation septic plasma or Ago2 monoclonal-treated septic plasma for further studies.

### 2.6. Enzyme-Linked Immunosorbent Assay (ELISA)

Plasma Ago2 levels were measured with commercial ELISA kits following the manufacturer's instructions (Lifespan).

### 2.7. Real-Time qPCR

Real-time qPCR (RT-PCR) assay was carried out to quantitate the miR-21a-3p and Nrp-1 mRNA levels as described before. In short, total RNAs from either plasma or cells were collected and purified according to the manufacturer's instructions (Invitrogen). First-Strand cDNA Synthesis Kit (TOYOBO) was used to synthesize cDNA while StepOne Real-Time PCR System (Invitrogen) was used to perform the relative real-time qPCR with Thunderbird SYBR qPCR Mix (TOYOBO). The U6 and GAPDH were used as internal control, respectively. The specific RNA levels were determined by the *ΔΔ*Ct method. The specific primers of the target RNAs and internal controls were as follows: miR-21a-3p: loop primer 5′-GTCGTATCCAGTGCAGGGTCCGAGGTATTCGCACTGGATACGACGACAGCCC -3′ and forward primer 5′- TGCGCCAACAGCAGTCGATGGG-3′; Nrp-1: forward primer 5′- ATTTCAAGTGTATGGAGGCT-3′ and reverse primer 5′- AGTAACGAATCGCAGGAG-3′; U6: forward primer 5′- CGCTTCGGCAGCACATATAC -3′ and reverse primer 5′- AAATATGGAACGCTTCACGA -3′; and GAPDH: forward primer 5′- ACAGCAACAGGGTGGTGGAC -3′ and reverse primer 5′- TTTGAGGGTGCAGCGAACTT -3′.

### 2.8. Western Blot Assay

The target protein expression levels were examined using Western Blotting as described previously. For TECs derived from the kidney of CLP rats, protease inhibitor containing RIPA buffer (Sigma) was used to extract the total protein. For samples derived from NRK52E in vitro, membrane proteins and cytoplasm proteins were firstly separated with Subcellular Protein Fractionation Kit for Cultured Cells (Thermo Scientific) in accordance with the manufacturer's instructions; then, the proteins from either membrane or cytoplasm were prepared with protease inhibitor containing RIPA buffer (Sigma). The protein lysates were separated with SDS-PAGE and transferred to the PVDF films (Millipore). The PVDF films then were cut into pieces according to the molecular weights of the target proteins and incubated with the primary antibodies (Ago2 rabbit monoclonal antibody, 1 : 1000 (Novus); Nrp-1 rabbit monoclonal antibody, 1 : 1000 (Novus); and His-tag mouse monoclonal antibody, 1 : 1000 (MBL)) at 4°C for 12 h. Then, either AlexaFluor 680/790-labeled goat anti-rabbit IgG antibody (1 : 10000, LI-COR Biosciences) or AlexaFluor 680/790-labeled goat anti-mouse IgG antibody (1 : 10000, LI-COR Biosciences) was used as the second antibody, and the blots were visualized by LI-COR Odyssey Infrared Imaging System (LI-COR Biosciences). Quantity One was used to quantify the blots.

### 2.9. Immunofluorescent Assay

The immunofluorescent assay was carried out in the present study as described before. In brief, the cell climbing films were fixed in 4% paraformaldehyde with 0.1% Triton X-100 for 30 min at 4°C. Relevant primary antibodies (Ago2 rabbit monoclonal antibody, 1 : 200 (Novus); Nrp-1 rabbit monoclonal antibody, 1 : 200 (Novus)) and fluorescently labeled probe (LysoTracker Red DND-99 (YeasenBio)) were used to incubate with the films overnight at 4°C according to the manufacturers' instructions. The films then were incubated with selected secondary antibodies (FITC-conjugated goat anti-rabbit IgG antibody and TRITC-conjugated goat anti-rabbit IgG antibody) specifically for 90 min in the dark. The nuclei were stained using 4′,6-diamidino-2-phenylindole. The images were captured using a laser confocal microscope (Olympus).

### 2.10. Flow Cytometry Assay

The flow cytometry assay was carried out to measure either cell membrane Nrp-1 level or cytoplasm Ago2 level. A Rat Neuropilin-1 PE-conjugated antibody (R&D) was used to determine the cell membrane Nrp-1 level by flow cytometry according to the manufacturer's instruction. For the cytoplasm Ago2 measurement, Fixation/Permeabilization Solution Kit (BD) was used to permeabilized the cells accordingly; then, Ago2 mouse monoclonal antibody (Novus) and FITC-conjugated goat anti-mouse IgG antibody (Boster) were used to determine the cytoplasm Ago2 level with flow cytometry. The cytometry was executed with Beckman CytoFLEX FCM.

### 2.11. Immunoprecipitation

Immunoprecipitations were performed according to the manufacturers' instructions. In short, the samples were incubated with the anti-Ago2 mouse monoclonal antibody (Novus) at 4°C overnight with shaking. Then, the mixtures were loaded with 40 *μ*l protein A+G agarose (Millipore) and incubated for 3 hours at 4°C. After that, the beads were collected with centrifugation and analyzed with immunoblotting for both His tag at 99 Kd and Nrp-1.

### 2.12. RNA Immunoprecipitation (RIP)

The Magna RIP™ RNA-Binding Protein Immunoprecipitation Kit (Millipore) was used to perform RIP assay according to the instructions. Ago2 rabbit monoclonal antibody (Novus) was used as the primary antibody, SNRNP70 rabbit polyclonal antibody (Invitrogen) was used as positive control while normal rabbit IgG was used as a negative control (ProteinTech). The immunoprecipitated RNA was isolated, and RT-PCR was used to analyze the enrichment of miR-21a-3p.

### 2.13. RNA Pull-down

miR-21a-3p and related controls were biotinylated by GeneParma. For TECs, the biotinylated oligonucleotides were incubated with TECs for 12 hours and the cells were lysed. For recombinant Nrp-1 (Abcam), the equimolar biotinylated oligonucleotides were added into the recombinant Nrp-1 PBS solution with or without recombinant Ago2 (SinoBiological) at the equimolar concentration. Subsequently, the samples were incubated with streptavidin-coated magnetic beads and biotin-coupled RNA complexes were pulled down. The relevant proteins were analyzed with immunoblotting.

### 2.14. Statistical Analysis

Quantitative data were presented as the mean ± SDs, and Graphpad Prism 8 was used to execute the statistical analysis. The means of different groups were analyzed using Student's *t*-test or one-way analysis of variance, followed by Tukey's multiple comparisons test. A *P* value < 0.05 was considered statistically significant.

## 3. Results

### 3.1. Both the miR-21a-3p and Ago2 Levels Increased in Plasma and TECs during Sepsis

**Figure 1:**
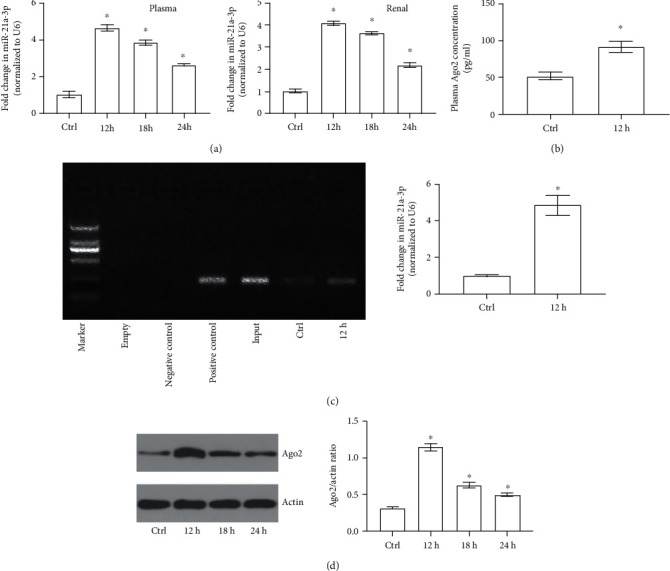
miR-21a-3p and Ago2 levels of both TECs and plasma during sepsis. (a) Quantitative analysis of RT-PCR results of miR-21a-3p of either plasma or TECs at different time points. (b) Quantitative analysis of ELISA for Ago2 concentrations of plasma at different groups. (c) Representative RIP results of Ago2 binding miR-21a-3p in plasma of different groups. (d) Representative Western Blot results of Ago2 in TECs of the control group and CLP groups at different time points (^∗^*P* < 0.05 vs. the control; Ctrl: control; 12 h: 12 hours after CLP; 18 h: 18 hours after CLP; 24 h: 24 hours after CLP).

To identify whether miR-21a-3p increased in both plasma and TECs, the CLP sepsis rat model was used in the present study. It can be found out with RT-PCR that both the miR-21a-3p levels in plasma and TECs increased significantly 12 h after the CLP procedure (Figure 1(a)). Because it was well studied that sepsis was induced 12 h after CLP in rats in our former studies [19], the present results indicated that the miR-21a-3p levels in both plasma and TECs were increased significantly during sepsis. Ago2 was known as one of the most important binding proteins of miRs in circulation and miRs were stabilized only if they bind to Ago2 or were present in exosomes in circulation. To further identify whether plasma Ago2 did also rise during sepsis, plasma Ago2 ELISA test was carried out in our present study. It can be demonstrated from the results that plasma Ago2 increased remarkably 12 h after CLP in sepsis rats (Figure 1(b)). Moreover, with RNA Binding Protein Immunoprecipitation Assay, it can be found out that miR-21a-3p could bind to Ago2 to form Ago2-miR-21a-3p complex and plasma Ago2 binding miR-21a-3p increased significantly as the plasma Ago2 level rose (Figure 1(c)). Furthermore, with Western Blot measurement, it was determined that the Ago2 level was remarkably upregulated in the TECs of CLP rats during sepsis (Figure 1(d)). The findings above suggested that the rise of miR-21a-3p and Ago2 in TECs may be closely related to the increase of Ago2 binding miR-21a-3p in plasma during sepsis.

### 3.2. Effects of Different Plasma Stimulation on the miR-21a-3p and Ago2 Levels in TECs

**Figure 2:**
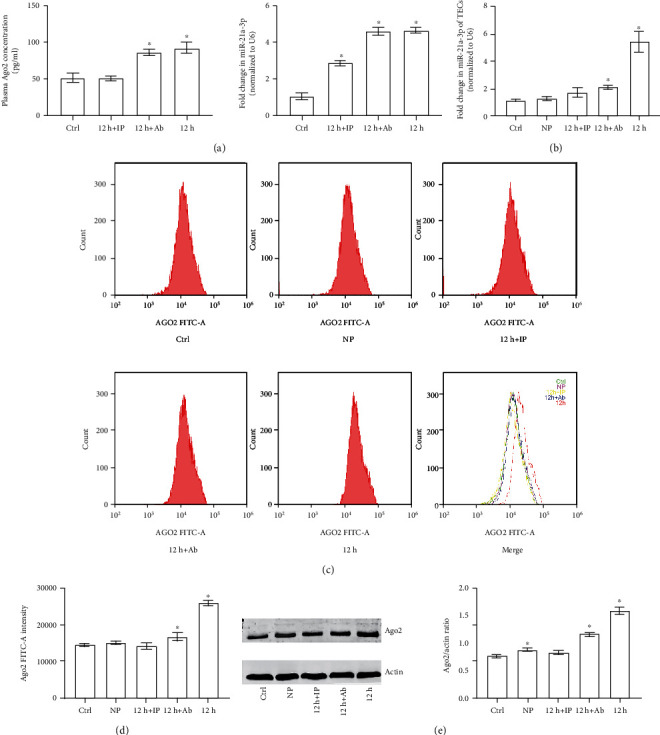
Ago2 and miR-21a-3p levels in TECs stimulated with differently treated plasma. (a) Quantitative analysis of the results from ELIA for Ago2 and RT-PCR for miR-21a-3p of plasma with or without treatments. (b) Quantitative analysis of RT-PCR results of miR-21a-3p of TECs treated with different plasma. (c) Representative results of flow cytometry for Ago2 in TECs treated with different plasma. (d) Quantitative analysis of the results of flow cytometry for Ago2 in TECs treated with different plasma. (e) Representative Western Blot results of Ago2 in TECs treated with different plasma (^∗^*P* < 0.05 vs. the control; Ctrl: control; NP: normal plasma; 12 h: septic plasma from rats 12 hours after CLP; 12 h+Ab: septic plasma treated with Ago2 antibody; 12 h+IP: septic plasma treated with Ago2 immunoprecipitation).

To further investigate the effects of septic plasma on the miR-21a-3p level in TECs and the role that plasma contained Ago2 plays in this process, we firstly obtained septic plasma from CLP rats at 12 h after the operation. After that, septic plasma was treated with either Ago2 monoclonal antibody (12 h+Ab) or Ago2 immunoprecipitation (12 h+IP) to regulate the activity and concentration of plasma Ago2 and Ago2 binding miR-21a-3p. It can be found out from the results that Ago2 immunoprecipitation significantly downregulated the concentrations of both plasma Ago2 and Ago2 binding miR-21a-3p. Ago2 monoclonal antibody did not affect the concentration of either Ago2 or Ago2 binding miR-21a-3p, but the monoclonal antibody would affect the biological activity of Ago2 in the plasma (Figure 2(a)). After that, TECs were treated with different kinds of plasma (control plasma: Ctrl; normal plasma: NP; septic plasma: 12 h; septic plasma treated with Ago2 immunoprecipitation: 12 h+IP; and septic plasma treated with Ago2 monoclonal antibody: 12 h+Ab). With RT-PCR, it could be found out that miR-21a-3p level of TECs in both the 12 h+Ab group and 12 h group increased significantly compared to the Ctrl and NP groups but the miR-21-3p level of the 12 h+Ab group was remarkable lower than that of the 12 h group, and the control and 12 h+IP groups exhibited similar miR-21a-3p levels (Figure 2(b)). Moreover, it was indicated by flow cytometry that only the Ago2 levels of both the 12 h and 12 h+Ab groups increased significantly compared to that of the Ctrl group and the Ago2 level of 12 h group was significantly higher than that of the NP and 12 h+Ab groups; the control, 12 h+IP, and NP groups exhibited similar levels of Ago2 (Figures 2(c) and 2(d)). In addition, Western Blot was carried out to identify the effects of different plasma stimulation on intracellular Ago2 levels in TECs. It can be verified from the results that the Ago2 levels of the NP, 12 h+Ab, and 12 h groups rose remarkably than the Ctrl and 12 h+IP groups and the intracellular Ago2 of the 12 h group was remarkably higher than that of the NP and 12 h+Ab groups; the intracellular Ago2 level did not change remarkably in the 12 h+IP group compared to the control (Figure 2(e)). Taken together, the results above indicated that septic plasma stimulation increased both the intracellular Ago2 and miR-21a-3p levels in TECs, while Ago2 in septic plasma was crucial for this process. Because a previous study had indicated that Ago2-mediated miR internalization, it was important to find out whether TECs could actively internalize miR-21a-3p and Ago2 from plasma during sepsis.

### 3.3. Effects of Septic Plasma Stimulation on Nrp-1 Expression and Distribution in TECs

**Figure 3:**
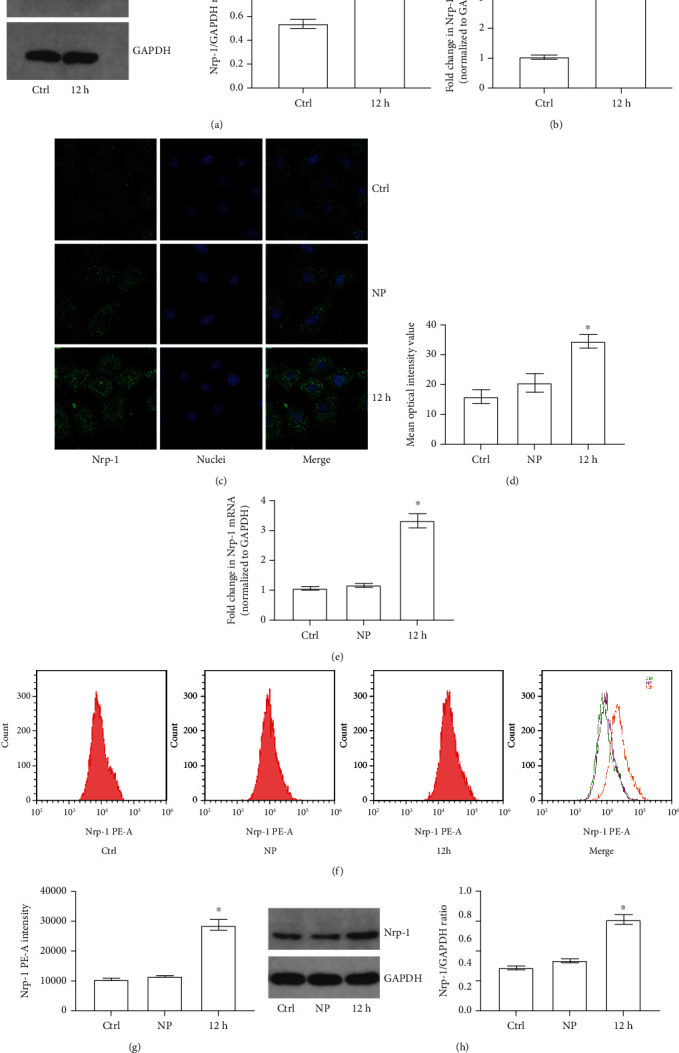
Nrp-1 expression and distribution of TECs in different groups. (a) Representative Western Blot results of Nrp-1 in TECs of either control or sepsis (12 h: 12 hours after CLP) rat model). (b) Quantitative analysis of RT-PCR results of Nrp-1 mRNA transcription level in TECs of either control or sepsis (12 h: 12 hours after CLP) rat model). (c) Representative fluorescent results of Nrp-1 expression and distribution of TECs treated with different plasma. (d) Quantitative analysis of fluorescent results of Nrp-1 expression. (e) Quantitative analysis of RT-PCR results of Nrp-1 mRNA transcription level in TECs treated with different plasma. (f) Representative flow cytometry results of membrane Nrp-1 on TECs treated with different plasma. (g) Quantitative analysis of flow cytometry results of membrane Nrp-1 on TECs treated with different plasma. (h) Representative Western Blot results of membrane Nrp-1 on TECs treated with different plasma (^∗^*P* < 0.05 vs. the control; Ctrl: control; NP: normal plasma; 12 h: septic plasma from rats 12 hours after CLP).

Nrp-1, a cell membrane receptor, is involved in the Ago2-mediated internalization of miRs. To find out whether miR-21a-3p could be internalized by TECs via the Ago2 pathway, it was important to verify the expression and distribution of Nrp-1 during sepsis. In vivo, our results of Western Blot and RT-PCR indicated that both the Nrp-1 expression and Nrp-1 mRNA transcription increased significantly in TECs during sepsis (Figures 3(a) and 3(b)). Moreover, an in vitro study was carried out with TECs stimulated with either normal plasma (NP group) or septic plasma (12 h group). Confocal microscopy analysis revealed that the expression of Nrp-1 in the 12 h group was higher than that in the NP and control groups (Figures 3(c) and 3(d)), and the Nrp-1 mRNA transcription level in the septic plasma group was also remarkable higher (Figure 3(e)). Furthermore, to clarify whether the increased Nrp-1 was distributed on the cell membrane, flow cytometry and Western Blot of the cell membrane protein were carried out. It could be determined from the results that cell membrane Nrp-1 increased significantly in TECs of the 12 h group than that of both the control group and the NP group (Figures 3(f)–3(h)). The results above indicated that the cell membrane Nrp-1 expression in TECs was increased dramatically during sepsis.

### 3.4. Plasma from Rats with Sepsis Enhances the Intracellular Ago2 and miR-21a-3p Levels through Nrp-1

**Figure 4:**
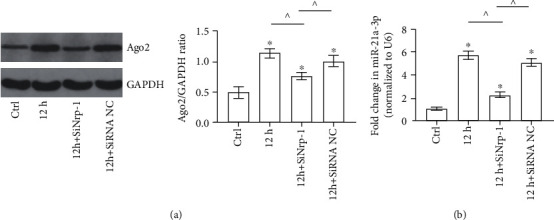
Ago2 and miR-21a-3p levels in TECs with or without Nrp-1 knockdown treated with septic plasma. (a) Representative Western Blot results of cytoplasm Ago2 of TECs with different treatments. (b) Quantitative analysis of RT-PCR results of miR-21a-3p of TECs with different treatments (^∗^*P* < 0.05 vs. the control, ^*P* < 0.05 between the two groups; Ctrl: control; 12 h: septic plasma from rats 12 hours after CLP; SiNrp-1: Nrp-1 siRNA transfection; SiRNA NC: siRNA negative control transfection).

To further clarify the role of Nrp-1 in the elevation of intracellular Ago2 and miR-21a-3p in TECs stimulated by septic plasma, septic plasma was used to incubate with normal TECs (12 h group), siRNA NC-transfected TECs (12 h+SiRNA NC group) and Nrp-1 siRNA-transfected TECs (12 h+SiNRP-1 group) and relevant tests had been carried out. With the Western Blot of cytoplasm, it can be demonstrated that intracellular Ago2 level was increased significantly in the 12 h group than that in the control group. Moreover, Nrp-1 knockdown mitigated the increase of intracellular Ago2 of the TECs incubated with septic plasma remarkably. The intracellular level of Ago2 in 12 h+SiNrp-1 group was still remarkable higher than that of the control group. There was no significant difference in the content of intracellular Ago2 in the TECs of the 12 h groups and 12 h+SiRNA NC group (Figure 4(a)). Furthermore, with RT-PCR, it can be verified that miR-21a-3p contents in TECs of the 12 h group, 12 h+SiNrp-1 group, and 12 h+SiRNA NC group were increased significantly than the control. The miR-21a-3p contents in TECs of the 12 h group and 12 h+SiRNA NC group were not significantly different and both were higher than that in TECs of the 12 h+SiNrp-1 group (Figure 4(b)). Taken together, the results above demonstrated that Nrp-1 played an important role in the elevation of intracellular Ago2 and miR-21a-3p in TECs stimulated with septic plasma.

### 3.5. Ago2 Was Internalized by TECs via Nrp-1 and This Process Had no Effect on the Intracellular Content of miR-21a-3p

**Figure 5:**
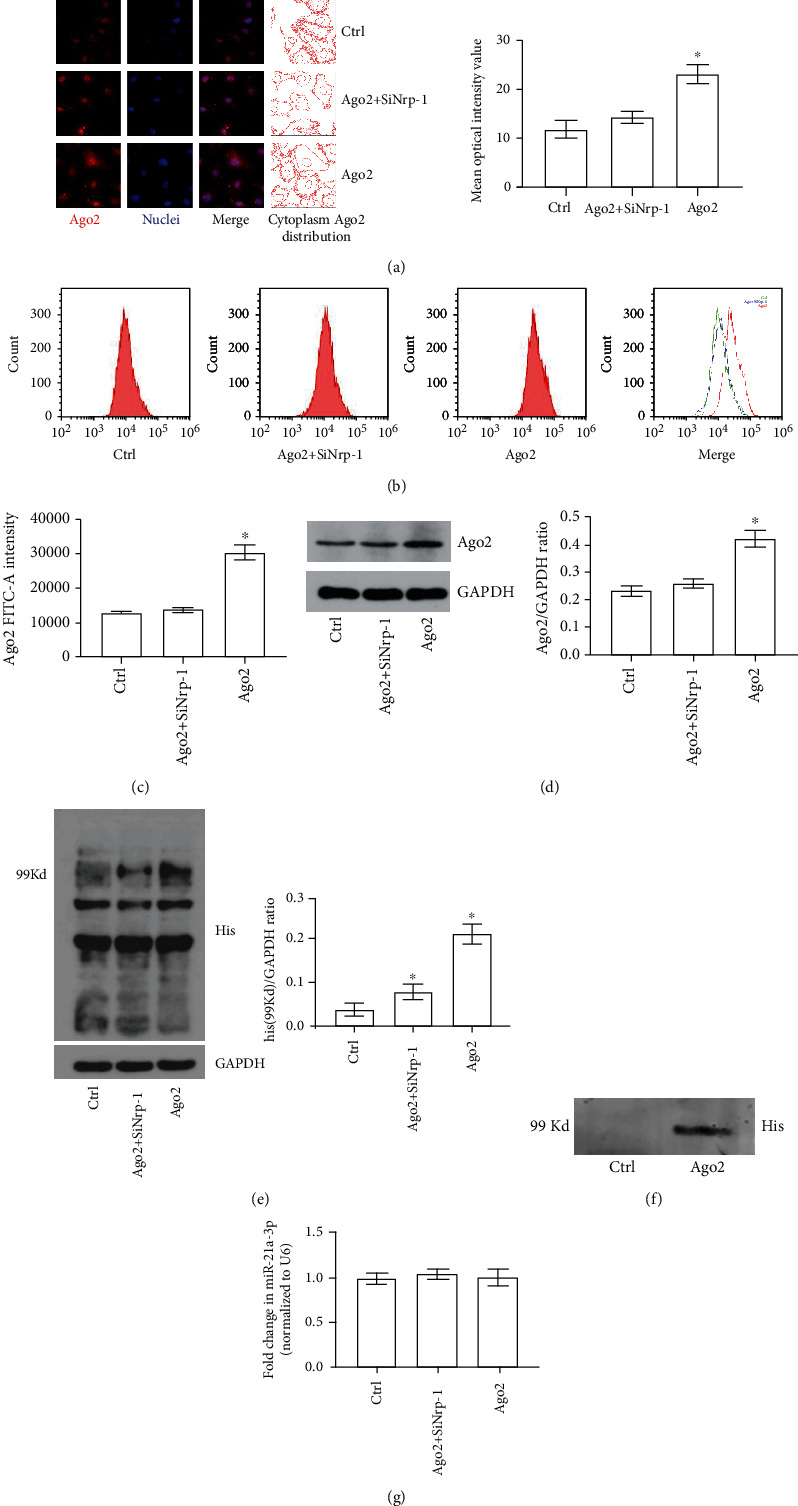
Exogenous Ago2 in TECs with different treatments. (a) Representative fluorescent results of cytoplasm Ago2 of TECs with different treatments. (b) Representative flow cytometry results of Ago2 in TECs with different treatments. (c) Quantitative analysis of flow cytometry results of Ago2 in TECs with different treatments. (d) Representative Western Blot results of cytoplasm Ago2 in TECs with different treatments. (e) Representative Western Blot results of cytoplasm His residue at 99 Kd in TECs with different treatments. (f) Representative results of immunoprecipitation of Ago2 binding His residue of TECs in the control and exogenous Ago2-treated groups. (g) Quantitative analysis of RT-PCR results of intracellular miR-21a-3p of TECs with different treatments (^∗^*P* < 0.05 vs. the control; Ctrl: control; SiNrp-1: Nrp-1 siRNA transfection).

To further verify whether Nrp-1 expressed by TECs could mediate the extracellular Ago2 internalization, His-tagged recombinant Ago2 was used to stimulate both the normal TECs (Ago2 group) and TECs with Nrp-1 knockdown by Nrp-1 siRNA (Ago2+SiNrp-1 group), after that, relative measurements were carried out in the present study. With immunofluorescent measurement, it can be found out that the cytoplasm Ago2 content increased significantly in the Ago2 group but not that in the Ago2+SiNrp-1 group compared to the control (Figure 5(a)). Flow cytometry also confirmed that cellular Ago2 content only rose remarkably in the Ago2 group compared to the control (Figures 5(b) and 5(c)). Moreover, Western Blot of cytoplasm proteins was carried out and it can be verified from the results that the TECs cytoplasm Ago2 of the Ago2 group increased significantly compared to the control and this phenomenon can be mitigated with Nrp-1 knockdown (Figure 5(d)). The above results indicated that Nrp-1 mediated the intracellular Ago2 content of TECs increasing when stimulated with extracellular Ago2. To further clarify whether the increased cytoplasm Ago2 was derived from Nrp-1 mediated cell internalization or cell synthesis, Western Blot and immunoprecipitation for His-tag of cytoplasm were carried out. It can be verified from the results that both the His-tag contents at 99 Kd of the Ago2 and Ago2+SiNrp-1 groups were increased significantly compared to the control. Moreover, the His-tag content at 99 Kd of the Ago2 group was remarkably higher than that of the Ago2+SiNrp-1 group (Figure 5(e)). Moreover, it can be found out that only in the TECs of the Ago2 group can His tag be detected by immunoblotting after cytoplasm Ago2 was immunoprecipitated with its monoclonal antibody (Figure 5(f)). Because the molecular weight of His-tagged extracellular Ago2 is 99 Kd, it can be verified that the increased cytoplasm Ago2 was derived from Nrp-1 mediated cell internalization. After all, with RT-PCR, it can be found out that only the Ago2 stimulation did not change the intracellular miR-21a-3p content no matter if Nrp-1 was knocked down or not (Figure 5(g)). Taken together, the results above indicated that Nrp-1 mediated the extracellular Ago2 internalization and this process did not affect the intracellular content of miR-21a-3p in TECs.

### 3.6. Both Ago2 and miR-21a-3p Could Bind to TEC-Derived Nrp-1 Directly

**Figure 6:**
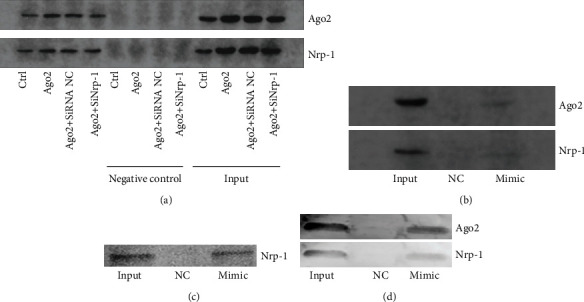
Interaction of Nrp-1 with either Ago2 or miR-21a-3p. (a) Representative results of immunoprecipitation of the cell lysates of TECs with different treatments; the lysates were immunoprecipitated with Ago2 antibody and probed with Ago2 and Nrp-1 antibodies after electrophoresis. (b) Representative results of RNA pull-down assay for lysates of TECs treated with biotin-miR-21a-3p single strain mimics. Biotin-miR-21a-3p was precipitated; Ago2 and Nrp-1 were probed with immunoblotting. (c) Representative results of RNA pull-down assay for biotin-miR-21a-3p/Nrp-1 mixture. Biotin-miR-21a-3p was precipitated and Nrp-1 was probed with immunoblotting. (d) Representative results of RNA pull-down assay for biotin-miR-21a-3p/Ago2/Nrp-1 mixture. Biotin-miR-21a-3p was precipitated; Ago2 and Nrp-1 were probed with immunoblotting (Ctrl: control; SiNrp-1: Nrp-1 siRNA transfection; SiRNA NC: siRNA negative control transfection; NC: miR-21a-3p mimic negative control; Mimic: miR-21a-3p mimics).

To further verify whether TEC-derived Nrp-1 could mediate the internalization of Ago2 as well as the Ago2 binding miR-21a-3p in TECs, it is crucial to find out whether TEC-derived Nrp-1 could interact with Ago2 and miR-21a-3p directly. Firstly, immunoprecipitation was carried out with lysates from different groups of TECs, the results suggested that TEC-derived Nrp-1 did bind to Ago2 directly (Figure 6(a)). Moreover, TECs were stimulated with biotin-labeled miR-21a-3p mimic and RNA pull-down assay was carried out. It can be suggested from the results that miR-21a-3p could interact with both the TEC-derived Ago2 and Nrp-1 (Figure 6(b)). To further verify whether miR-21a-3p could directly bind to Nrp-1 derived from TECs, biotin-labeled miR-21a-3p mimic was mixed with either recombinant rat Nrp-1 or recombinant rat Nrp-1/Ago2 mixture. It can be verified from the results of the RNA pull-down assay that miR-21a-3p did interact with the Nrp-1 directly regardless of the presence of Ago2 (Figures 6(c) and 6(d)). In summary, the above results indicated that both Ago2 and miR-21a-3p could bind to TEC-derived Nrp-1 directly.

### 3.7. miR-21a-3p Was Internalized by TECs via Nrp-1 and Ago2 Facilitated This Process

**Figure 7:**
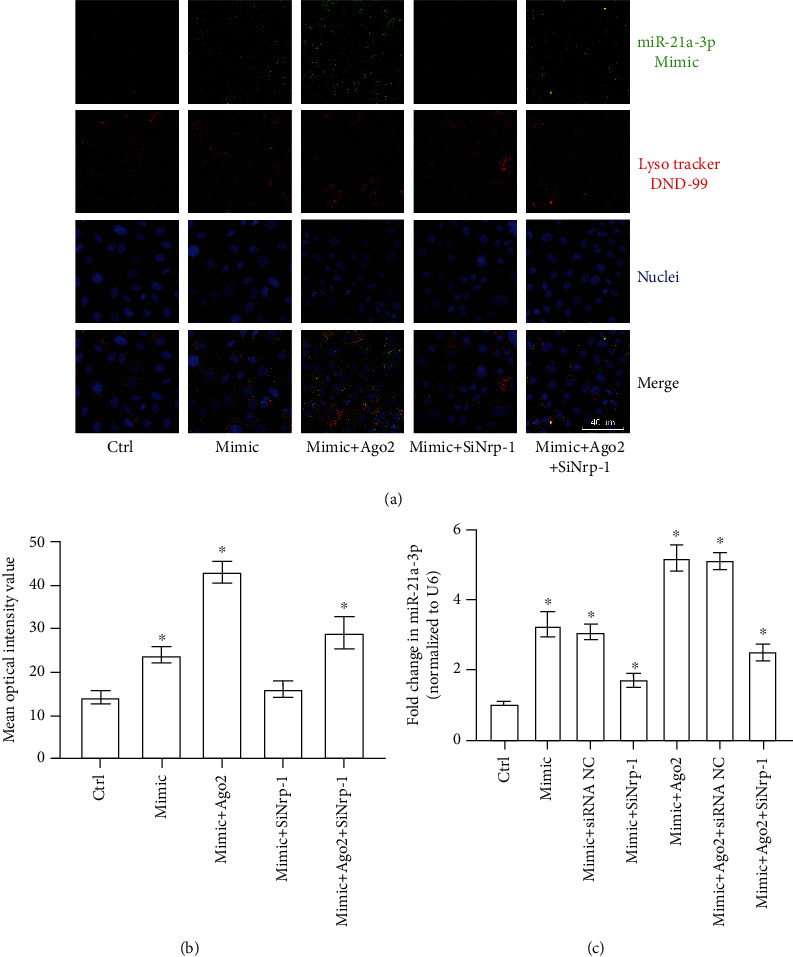
miR-21a-3p in TECs with different treatments. (a) Representative fluorescent results of intracellular FAM-miR-21a-3p mimics in TECs with different treatments. (b) Quantitative analysis of FAM optical intensities of fluorescent results of intracellular FAM-miR-21a-3p mimics in TECs with different treatments. (c) Quantitative analysis of RT-PCR results of miR-21a-3p in TECs with different treatments. (^∗^*P* < 0.05 vs. the control; Ctrl: control; SiNrp-1: Nrp-1 siRNA transfection; SiRNA NC: siRNA negative control transfection; Mimic: miR-21a-3p mimics).

Since miR-21a-3p could interact with TEC-derived Nrp-1, it was important to clarify if miR-21a-3p could be internalized by TECs and what the roles of Nrp-1 and Ago2 play in this process. To answer the above questions, TECs were divided into several groups based on whether or not Nrp-1 knockdown was performed and either FAM-miR-21a-3p mimics or Ago2/FAM-miR-21a-3p mimics complex was used to incubate with the different groups of TECs. Firstly, confocal microscopy was carried out to measure the cytoplasm FAM-miR-21a-3p content, while lysosomes were marked with Lyso-tracker to visualize the cytoplasm. It can be found out from the immunofluorescence results that cytoplasm FAM optical intensities were both increased significantly when TECs were incubated with either FAM-miR-21a-3p or Ago2/FAM-miR-21a-3p complex. Moreover, FAM optical intensity in TECs incubated with Ago2/FAM-miR-21a-3p complex was remarkably higher than that in TECs incubated only with FAM-miR-21a-3p. Nrp-1 knockdown could significantly mitigate this phenomenon (Figures 7(a) and 7(b)). Furthermore, either miR-21a-3p mimics or Ago2/miR-21a-3p mimics complex was used to incubate with normal, siRNA negative control-transfected, or Nrp-1 siRNA-transfected TECs. It can be verified from the RT-PCR results that the intracellular miR-21a-3p contents were increased significantly in the TECs incubated with either miR-21a-3p mimics or Ago2/miR-21a-3p mimics complex than the control. Moreover, TECs incubated with Ago2/miR-21a-3p mimics complex had remarkable higher miR-21a-3p content than TECs incubated only with miR-21a-3p mimics. Nrp-1 knockdown could significantly suppress the increase of miR-21a-3p in both the TECs incubated with miR-21a-3p mimics and the TECs incubated with Ago2/miR-21a-3p mimics complex (Figure 7(c)). According to the above results, it can be clarified that miR-21a-3p can be internalized by TECs via Nrp-1 and Ago2 facilitated this process.

## 4. Discussion

Sepsis is a global health concern as it causes millions of deaths each year. The prevalence of SAKI in patients admitted to ICU is 10–20%. Thus, sepsis is the most common cause of AKI in critically ill patients. Compared with that among patients without AKI, the mortality rate is 50% higher among patients with SAKI [26]. The mechanisms underlying SAKI have not been elucidated. TECs act as one of the most vulnerable inherent cells in the kidney during sepsis. It has been demonstrated that when stimulated with sepsis-associated circulatory factors such as LPS and HMGB1, TECs will undergo a series of morphological and functional abnormalities which become the pivotal causes of AKI [27–29]. Moreover, our previous study has demonstrated that the miR-21a-3p content was increased in TECs, while miR-21a-3p mediates metabolism and cell fate alteration of TECs via manipulating the AKT/CDK2-FOXO1 pathway during SAKI [19]. In order to better understand the mechanisms of how miR-21a-3p induce TEC malfunction during inflammation, it is important to further reveal the source of increased intracellular miR-21a-3p of TECs during sepsis. It is well known that TECs are constantly in contact with blood and are regulated by various signaling molecules in plasma. A previous study also indicated that the composition of circulated microRNAs changed significantly during sepsis [30, 31]. Here, in our present study, the results of RT-PCR have demonstrated that plasma miR-21a-3p content was increased significantly when sepsis occurred. Moreover, consistent with our previous findings, our present study indicated that the intracellular miR-21a-3p content of TECs also rose remarkably as the plasma miR-21a-3p level increased during sepsis. Argonaute-2 (Ago2) is a highly conserved member of the Argonaute family in species and it has been revealed that small RNA-guided gene silencing can be regulated by Ago2 in humans [32]. Ago2 binds and stabilizes the miRs in the plasma. Previous studies have verified that microRNAs can only stably exist in circulation when forming complexes with Ago2 or HDL or are contained in exosomes [23, 33]. In our present study, it was revealed that the Ago2 content in both plasma and TECs rose remarkably and Ago2 binding miR-21a-3p increased significantly during sepsis. It has been demonstrated that Ago2 can mediate the internalization of mircoRNAs in previous studies [34, 35]. Therefore, we hypothesized that the increased internalization of plasma miR-21a-3p mediated by Ago2 could be an important mechanism mediated the rise of intracellular miR-21a-3p in TECs during sepsis.

Using in vitro cell lines for further study can eliminate the interference of in vivo confounding factors and be more accurate. In rats, sepsis is induced at 12 h post-CLP operation [19], and septic plasma from CLP rats at 12 h were used for the in vitro study. The monoclonal antibodies can bind to their target protein specifically and inhibit their biological activity. Additionally, it has been shown that immunoprecipitation can remove Ago2 out of solution or cell lysis effectively [36, 37] but may cause some nonspecific clearance. In our present study, septic plasma from CLP rats was treated with either Ago2 monoclonal antibody (Ago2+Ab) or Ago2 immunoprecipitation (Ago2+IP). Immunoprecipitation effectively decreased the levels of Ago2 and miR-21a-3p in the plasma. Treatment with anti-Ago2 monoclonal antibody did not significantly affect the levels of Ago2 and miR-21a-3p. However, the biological activity of Ago2 was inhibited upon treatment with the anti-Ago2 antibody. Furthermore, we stimulated the TECs of the NRK-52E cell line with normal rat plasma, septic plasma treated with Ago2 monoclonal antibody, septic plasma treated with Ago2 immunoprecipitation, and septic plasma. It was verified from the results that septic plasma could significantly increase the intracellular Ago2 and miR-21a-3p contents, while the reduction of Ago2 in septic plasma could suppress this phenomenon. It can be concluded from the results that Ago2 was the key factor in the rise of miR-21a-3p in TECs stimulated by septic plasma and that conclusion further supported our hypothesis that Ago2 may mediate the internalization of exogenous miR-21a-3p into TECs during sepsis.

There must be receptors expressed on the cell membrane of TECs which can mediate internalization so that miR-21a-3p could be internalized associated with Ago2. Neuropilin-1 (Nrp-1) is known to be the receptor that can bind various ligands via its extracellular part consisted of several domains [38]. The original study has shown that Nrp-1 was expressed by neurons as an adhesion receptor [39]. However, it has been clarified that not only neurons but also several other kinds of cells such as renal cells express Nrp-1 [40, 41]. Recent studies have indicated that Nrp-1 could implicate in the signal events of VEGF, TGF-*β*, and PDGF [42, 43]. Moreover, the number of Nrp-1-related signaling receptor complexes kept on growing. Nowadays, it has been reported that Nrp-1 was able to transport other extracellular molecules into cells as playing a cell “cargo” role [44]. It was demonstrated that Ago2 can interact with Nrp-1 and can be transported into cells mediated by Nrp-1. To further study whether Nrp-1 also acted as the receptor which can mediate the internalization of Ago2 and miR-21a-3p in TECs, it was pivotal to find out whether Nrp-1 was expressed by TECs, especially during sepsis. The location of which Nrp-1 was expressed during sepsis was also needed to be revealed. In our present study, it can be demonstrated from both the in vivo and in vitro results that Nrp-1 was expressed on the membrane of the TECs and that expression was enhanced when TECs were stimulated with the septic plasma during sepsis. Hence, we assumed that Nrp-1 expressed by TECs was the receptor that could mediate the internalization of miR-21a-3p as well as Ago2. To verify our assumption, firstly we need to find out the role that Nrp-1 plays in the rise of intracellular Ago2 and miR-21a-3p when TECs were stimulated with septic plasma. Here, in our present study, Nrp-1 siRNA was used to suppress the expression of Nrp-1 in TECs stimulated with septic plasma. It can be verified from the results that Nrp-1 knockdown significantly suppressed the increase of either Ago2 or miR-21a-3p content in the TECs stimulated with septic plasma. These results strongly suggested that Nrp-1 was crucial for the rise of intercellular Ago2 and miR-21a-3p. Hence, we hypothesized that Ago2 binding miR-21a-3p from septic plasma may be internalized by TECs via Nrp-1 during sepsis.

Next, to further verify our hypothesis, it was important to find out whether Nrp-1 can mediate the internalization process of extracellular Ago2 and miR-21a-3p in TECs. First of all, to verify if exogenous Ago2 could be internalized by TECs via Nrp-1, His-tagged recombinant Ago2 was used to incubate with either normal TECs or Nrp-1 knockdown TECs and relative measurements were carried out in the present study. It can be demonstrated from the results that Nrp-1 was crucial for the increase of intercellular Ago2 in TECs incubated with His-tagged recombinant Ago2. However, these findings do not indicate if the increased Ago2 in the cells is due to the internalization of exogenous Ago2 or the active syncretization of the cells after being stimulated. His tag is an amino acid residue consisting of 6 histidines, and it is one of the most common tags used to facilitate the purification of recombinant proteins [45]. His tag monoclonal antibody is used to monitor the His-tagged proteins, and this monoclonal antibody can recognize His tags placed at the N-terminal, C-terminal, and internal regions of the recombinant proteins. Because the recombinant Ago2 we used in the present study was labeled with His tag and the molecular weight of Ago2 was about 99 Kd, Western Blot was used to verify the cytoplasm content of His residue illustrated at 99 Kd in the TECs of different groups. It was demonstrated from the results that although Ago2 was expressed in normal TECs, the His residue appeared at 99 Kd only can be found in the TECs incubated with His-tagged Ago2. Moreover, Nrp-1 knockdown in TECs could significantly mitigate this phenomenon. To further verify whether the His residue at 99 Kd found with Western Blot was from the His-tagged Ago2, immunoprecipitation of His residue with Ago2 monoclonal antibody was carried out and the results indicated that it was the His-tagged Ago2 that was visualized by His monoclonal antibody at 99 Kd when tested with Western Blot. Taken together, it can be concluded that Nrp-1 mediated the internalization of the exogenous Ago2 in TECs. Furthermore, we found out that both Ago2 stimulation and internalization via Nrp-1 did not change the intercellular miR-21a-31p level in TECs. Hence, next was to find out whether exogenous miR-21a-3p could be internalized via Nrp-1 in TECs and the role Ago2 played in this process.

Previous studies have demonstrated that Nrp-1 was the receptor for receptor-mediated cell internalization of many exogenous molecules such as VEGF in neurons and tumor cells [46, 47]. For the occurrence of receptor-mediated cell internalization, ligands must be able to bind to the receptors directly. In our present study, Ago2 was determined to be able to bind Nrp-1 directly with a coimmunoprecipitation test. Hence, consistent with the findings of previous studies, Nrp-1 was demonstrated to function as a cell membrane receptor for the internalization of exogenous Ago2 into the TECs. Moreover, miRs are stable in the plasma when they bind to Ago2 or are enclosed in the exosomes, while only the Ago2 binding miRs can be internalized into cells via a receptor-mediated cell internalization pathway. Hence, to verify whether miR-21-3p could be internalized via Nrp-1, it was also important to find out if miR-21a-3p could bind to Nrp-1 in the presence or absence of Ago2. With RNA pull-down assay, we demonstrated that miR-21a-3p can bind to TEC-derived Nrp-1 directly even in the absence of Ago2. Therefore, the results mentioned above provided a strong biological basis for our hypothesis. Finally, miR-21a-3p single-strain mimics with or without FAM label were used to examine the Nrp-1-mediated internalization of miR-21a-3p into the TECs and the role of Ago2 in this process. Our results indicated that miR-21a-3p can be internalized by TECs via Nrp-1 and Ago2 facilitated this process.

To the best of our knowledge, this is the first study to demonstrate that Ago2 binding miR-21a-3p from septic plasma can be actively internalized by TECs via Nrp-1-mediated cell internalization, which enhances the intracellular miR-21a-3p levels in the TECs during sepsis. Although further researches are needed to verify some unsolved questions such as (1) through which specific pathway that Ago2-miR-21a-3p enters the TECs under the mediation of Nrp-1, (2) what other cellular mechanisms can mediate this process; (3) does exosome miR-21a-3p also participate in the rise of intracellular miR-21a-3p in TECs during sepsis; (4) does the mechanism elucidated in this study also exist in other cell types; and (5) is the internalization mechanism of miR-21a-3p also applicable to other miRs in TECs. For miR-21a-3p can manipulate cell metabolisms of TECs during sepsis, which is one of the most important mechanisms that induced SAKI, our findings will provide a novel basis for understanding the mechanisms underlying SAKI and aid in developing new relative therapeutic strategies accordingly.

## 5. Conclusions

The internalization of plasmatic Ago2 binding miR-21a-3p mediated by membrane Nrp-1 is an important mechanism underlying miR-21a-3p accumulation in TECs during sepsis.

## Data Availability

The data used to support the findings of this study are either included within the article or available from the corresponding author upon request.
